# Crosstalk between endoplasmic reticulum stress and mitochondrial homeostasis: A new perspective on ophthalmic disease treatment

**DOI:** 10.1002/ccs3.70080

**Published:** 2026-05-16

**Authors:** Luyang Jiang, Gongsang Cuomu, Sang Jijia, Yumei Yang, Jufen Liu, Yumin Tao

**Affiliations:** ^1^ Eyes Center Shangyu People's Hospital of Shaoxing Shaoxing University Shaoxing Zhejiang China; ^2^ School of Medicine Shaoxing University Shaoxing Zhejiang China; ^3^ Department of Ophthalmology People's Hospital of Biru County Nagqu City China; ^4^ The Affiliated Kangning Hospital of Ningbo University Ningbo Zhejiang Province China

**Keywords:** calcium signaling, endoplasmic reticulum–mitochondria crosstalk, mitochondria‐associated membranes, mitochondrial dynamics, mitophagy, ophthalmic diseases, unfolded protein response

## Abstract

Endoplasmic reticulum (ER) stress and mitochondrial dysfunction are hallmarks of many ophthalmic diseases; however, they have traditionally been examined as isolated pathological processes. Recent evidence indicates that these organelles are inextricably coupled through mitochondria–endoplasmic reticulum contact sites, also known as mitochondria‐associated membranes (MAMs), which coordinate Ca^2+^ signaling, lipid transfer, mitochondrial dynamics, redox balance, and cell death decisions. Consequently, dysregulated ER–mitochondria communication has emerged as a key vulnerability that links the cellular stress responses among diverse ocular tissues, including lens epithelial cells, retinal ganglion cells, the retinal pigment epithelium, and corneal endothelial cells. In this review, we summarize the recent advances involving the molecular architecture and regulatory function of ER–mitochondria crosstalk. We focus on how the unfolded protein response signaling, pathological MAM remodeling, Ca^2+^ dysregulation, and disrupted mitochondrial quality control collectively drive disease progression. By integrating evidence from cataract, glaucoma, diabetic retinopathy, age‐related macular degeneration, and Fuchs endothelial corneal dystrophy, we reveal that these disorders are not driven by a uniform mechanism of organelle failure, but rather by the dominance of pathological nodes along the ER–mitochondria axis. We propose that ophthalmic diseases should be stratified based on these distinct failure nodes, which provides a mechanistic framework for developing therapeutics. Within this context, interventions targeting maladaptive ER stress, MAM destabilization, bioenergetic failure, or defective mitophagy should be considered complementary and context‐dependent strategies. By reframing ophthalmic disorders as diseases of inter‐organelle stress integration, this review positions the ER–mitochondria axis as a modifiable upstream determinant of ocular cell fate, which provides a foundation for stage‐specific precision therapies.

## Graphical abstract

The graphical abstract follows a top‐to‐bottom flow: stressors → ER–mitochondria crosstalk → ophthalmic diseases. The central panel highlights three core mitochondria‐associated membrane function axes, color‐coded as follows: light yellow for Ca^2+^ homeostasis, light blue for mitochondrial dynamics, and light red for mitophagy. These colors extend to the disease panel (cataract, glaucoma, diabetic retinopathy, AMD, FECD) to visually map shared versus disease‐specific mechanisms. AMD represents age‐related macular degeneration; Ca^2+^, calcium ions; FECD, Fuchs endothelial corneal dystrophy.

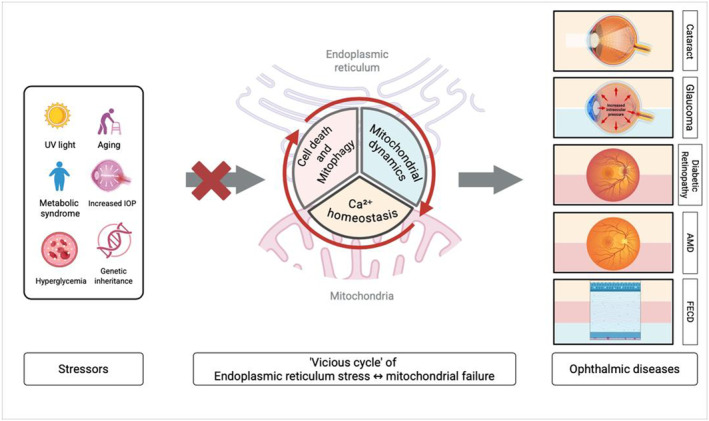

## 1 INTRODUCTION

Eukaryotic cells maintain homeostasis through highly coordinated communication among membrane‐bound organelles. Traditionally, studies of cellular stress responses have focused on individual organelles, such as the endoplasmic reticulum (ER) in protein folding or the mitochondria for energy production; however, these two organelles are not isolated entities. Instead, they engage in extensive physical and functional crosstalk mediated by specialized microdomains known as mitochondria–ER contact sites (MERCs) or mitochondria‐associated membranes (MAMs).^1^, ^2^, ^3^, ^4^ These contact sites regulate Ca^2+^ exchange, lipid transfer, mitochondrial dynamics, redox signaling, and apoptotic pathways, which indicate that ER–mitochondria communication is an important determinant of cell fate during stress.^3^, ^5^, ^6^, ^7^ Disruption of this coupling has been implicated in metabolic disorders, neurodegeneration, immune dysregulation, and aging‐related diseases.^8^

Ophthalmic tissues, including the lens epithelium, retinal ganglion cells (RGCs), retinal pigment epithelium (RPE), and corneal endothelium, are uniquely vulnerable to oxidative, metabolic, and proteotoxic stress because of their high energy requirements, lifelong light exposure, and limited regenerative capacity.^9^, ^10^, ^11^, ^12^ Under such conditions, misfolded proteins accumulate in the ER and activate the unfolded protein response (UPR). Although transient UPR activation restores proteostasis, sustained signaling through protein kinase RNA‐like endoplasmic reticulum kinase (PERK)–eukaryotic initiation factor 2 alpha (eIF2α)–activating transcription factor 4 (ATF4)–the C/EBP homologous protein (CHOP), inositol‐requiring enzyme 1α (IRE1α)/X‐box‐binding protein‐1 (XBP1), and activating transcription factor 6 (ATF6) induces apoptosis, inflammatory signaling, mitochondrial dysfunction, and metabolic reprogramming.^9^, ^13^, ^14^ Many UPR components, such as PERK, IRE1α, and chaperones (e.g., GRP75), are enriched at MAMs, where they regulate mitochondrial Ca^2+^ transfer, cristae integrity, and bioenergetic output.^15^, ^16^, ^17^ These results indicate that ER stress and mitochondrial failure in ocular cells are not parallel, but deeply interconnected processes.

Despite significant advances in elucidating ocular disease mechanisms, current treatment regimens primarily target downstream pathological manifestations rather than upstream organelle‐level stressors. Cataract treatment still involves surgery rather than the prevention of ER‐stress‐induced proteostasis collapse in the lens epithelium.^9^ In glaucoma, reducing intraocular pressure (IOP) remains the clinical standard, it does not necessarily ameliorate the early, organelle‐driven pathology, such as mitochondrial fragmentation and ER–mitochondria dysfunction, in RGCs.^18^, ^19^, ^20^ Similarly, anti‐vascular endothelial growth factor (VEGF) therapy for diabetic retinopathy (DR) and age‐related macular degeneration (AMD) does not target upstream ER stress, redox imbalance, and mitophagy failure.^9^, ^12^, ^21^

Dysregulated ER–mitochondria communication is an early and common pathological driver among these diseases,^12^, ^21^ which positions the ER–mitochondria axis as a promising therapeutic entry point. Regulating UPR branches (e.g., PERK, ATF6, IRE1α) reduces apoptosis and metabolic stress in lens, retinal, and corneal models.^22^, ^23^, ^24^, ^25^ Stabilizing MAM integrity and restoring Ca^2+^ transfer defects attenuate mitochondrial dysfunction in glaucoma and DR models.^20^, ^21^, ^26^, ^27^, ^28^ Enhancing mitochondrial quality control (QC), including PINK1/Parkin‐dependent mitophagy, protects against RPE, RGC, and lens epithelial cell (LEC) degeneration.^29^, ^30^, ^31^, ^32^ Finally, reinforcing mitochondrial antioxidant defenses reduces ER–mitochondria stress loops in DR, AMD, and Fuchs endothelial corneal dystrophy (FECD) models.^33^, ^34^

Because of the rapidly growing recognition of ER–mitochondria crosstalk as a central regulator of ocular cell survival, a comprehensive synthesis of this field is timely. This review summarizes the molecular architecture and regulatory mechanisms involved in ER–mitochondria communication, integrates recent evidence linking its dysfunction to major ophthalmic diseases, and highlights emerging treatment strategies targeting this inter‐organelle axis. Based on the current evidence, this review proposes that ophthalmic diseases can be mechanistically stratified by distinct failure nodes along the ER–mitochondria axis, which provides a conceptual framework for the development of mechanism‐based and stage‐specific therapeutics.

## 2 ER STRESS SIGNALING AS A DRIVER OF ER–MITOCHONDRIA CROSSTALK

The ER is a key organelle for cellular proteostasis, lipid synthesis, and Ca^2+^ storage. It enables cells to adapt to fluctuating metabolic and environmental demands. In ocular tissues, such as LECs, RGCs, RPE, and corneal endothelial cells (CECs), these functions are continuously challenged by oxidative stress, photo‐exposure, high metabolic demand, and limited regenerative capacity.^35^, ^36^ Consequently, disrupting ER homeostasis activates the UPR, which positions ER stress as an early and common initiator of cellular dysfunction in ophthalmic diseases.^9^, ^36^

In mammals, UPR signaling is mediated by three ER‐resident transducers (IRE1α, PERK, and ATF6), which collectively coordinate adaptive responses aimed at restoring proteostasis.^13^, ^37^, ^38^, ^39^ Although transient UPR activation is cytoprotective, sustained signaling shifts the balance toward apoptosis, inflammation, and metabolic reprogramming.^13^, ^40^ Importantly, the pathological consequences of prolonged ER stress are not confined to the ER itself, but propagate through direct physical and functional coupling with the mitochondria, thereby incorporating ER stress into a broader inter‐organelle stress network.^41^

### 2.1 UPR sensors at the ER–mitochondria interface

The recognition that UPR transducers exert non‐canonical functions at MAMs, where ER and mitochondrial membranes are apposed is a major advancement in this field.^17^, ^42^ At these contact sites, UPR components directly affect mitochondrial Ca^2+^ handling, bioenergetics, and cell‐fate decisions, linking ER proteostasis to mitochondrial function.^43^

Beyond its canonical role in XBP1 splicing, IRE1α acts as a structural regulator of ER–mitochondria contacts. Loss of IRE1α disrupts the spatial organization of inositol 1,4,5‐trisphosphate receptors (IP_3_Rs) at the MAMs, which results in impaired mitochondrial Ca^2+^ uptake and altered basal bioenergetics. These effects are independent of canonical UPR transcriptional outputs.^17^, ^44^, ^45^

PERK is also enriched at MAMs and contributes to adaptive and maladaptive ER–mitochondria signaling.^9^ Through interactions with mitochondrial tethering proteins, such as mitofusin‐2 (MFN2), PERK stabilizes ER–mitochondria contacts during stress conditions.^16^ Sustained PERK activation propagates oxidative stress and apoptosis through CHOP expression and disrupted Ca^2+^ homeostasis.^15^, ^46^

ATF6 is a transcriptional regulator of ER folding capacity and lipid metabolism; however, it has emerging roles in the epithelial–mesenchymal transition (EMT) and stress adaptation in ocular cells.^24^ These affect mitochondrial function indirectly by reshaping cellular metabolism and redox balance.

Taken together, these results support a model in which UPR sensors act not only as ER stress detectors, but also as functional nodes at ER–mitochondria interfaces, by translating the proteostasis imbalance into mitochondrial dysfunction during chronic stress.

## 3 STRUCTURAL AND FUNCTIONAL ORGANIZATION OF ER–MITOCHONDRIA CONTACT SITES

Mitochondria are dynamic organelles that interact with other intracellular compartments to regulate metabolism, signaling, and cell survival.^47^ Of these interactions, ER–mitochondria contact sites, also known as MAMs, are a central hub for inter‐organelle communication. MAMs comprise a small fraction of the mitochondrial surface, but exert disproportionate influence over Ca^2+^ signaling,^48^ lipid transfer,^49^ mitochondrial dynamics,^50^ and programmed cell death.^51^

### 3.1 Core molecular axes of MAM function

**FIGURE 1:**
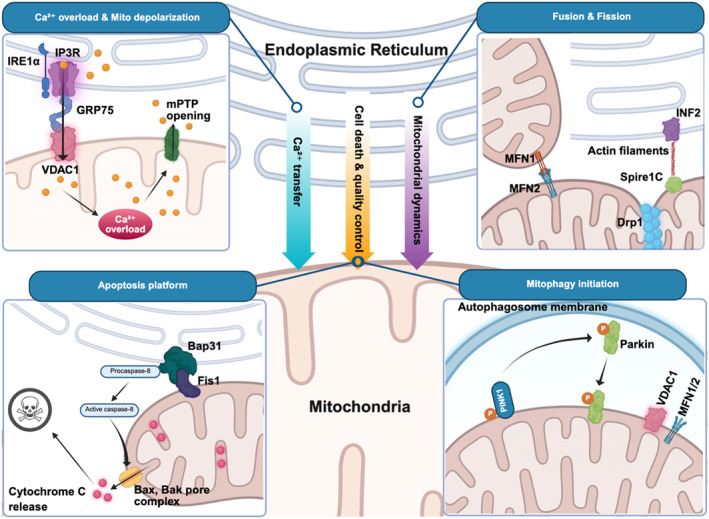
Structural and functional organization of ER–MAMs. MAMs are specialized contact sites between the ER and mitochondria that coordinate calcium (Ca^2+^) signaling, mitochondrial dynamics, and cell fate regulation. Ca^2+^ transfer (upper left): The IP_3_R–GRP75–VDAC1 complex forms a functional bridge enabling ER‐to‐mitochondria Ca^2+^ flux. IRE1α can localize to this interface and modulate Ca^2+^ signaling. While controlled Ca^2+^ transfer supports mitochondrial metabolism, excessive Ca^2+^ flux promotes mitochondrial Ca^2+^ overload, opening of the mitochondrial permeability transition pore, and membrane depolarization. Mitochondrial dynamics (upper right): ER–mitochondria contacts define sites of mitochondrial fission through ER‐associated actin remodeling (e.g., INF2) and recruitment of DRP1. MFN1/2 regulate fusion and contribute to ER–mitochondria tethering. Imbalance of fission and fusion under stress leads to mitochondrial fragmentation and functional decline. Apoptosis and mitophagy (lower panels): MAMs act as decision nodes for cell fate. The BAP31–FIS1 complex facilitates caspase activation, BAX/BAK pore formation, and cytochrome c release, promoting apoptosis. In parallel, MAMs provide platforms for mitophagy initiation via the PINK1–Parkin pathway, enabling removal of damaged mitochondria and maintenance of cellular homeostasis. Together, these interconnected axes position MAMs as central regulators that translate ER stress into mitochondrial structural and functional outcomes. Dysregulation of MAM signaling contributes to mitochondrial dysfunction and regulated cell death in ocular cells. BAK, Bcl‐2‐antagonist killer; BAX, Bcl‐2‐associated X protein; ER, endoplasmic reticulum; IRE1α; inositol‐requiring enzyme 1α; MAM, mitochondria‐associated membrane; MFN, mitofusin.

Instead of being uniform or static interfaces, MAMs are organized into discrete and functionally specialized molecular modules whose composition, spacing, and signaling output are dynamically regulated. Structural, biochemical, and imaging studies indicate that ER–mitochondria contacts can be divided into three interdependent, but mechanistically distinct axes: Ca^2+^ transfer, mitochondrial dynamics, and cell‐death/quality‐control signaling. Together, they determine mitochondrial fitness and cell fate under certain physiological and pathological conditions (Figure 1).

#### 3.1.1 Ca^2+^ transfer axis: Metabolic tuning versus Ca^2+^ toxicity

The efficient transfer of Ca^2+^ from the ER to the mitochondria at the MAMs is mediated by the IP_3_R–GRP75–VDAC1 macro‐complex, which physically and functionally couples ER Ca^2+^ release to mitochondrial Ca^2+^ uptake across the outer mitochondrial membrane. GRP75 (HSPA9) is a molecular chaperone that bridges IP_3_Rs to VDAC1, thereby ensuring the formation of high Ca^2+^ microdomains at contact sites that overcome the low affinity of the mitochondrial Ca^2+^ uptake machinery.^52^, ^53^ Ca^2+^ uptake by the mitochondria at these microdomains activates Ca^2+^‐sensitive dehydrogenases from the tricarboxylic acid cycle, such as pyruvate, isocitrate, and α‐ketoglutarate dehydrogenases. This provides a mechanistic basis for linking ER Ca^2+^ signaling to adenosine triphosphate (ATP) production and redox homeostasis^54^; however, this axis is exquisitely dose‐dependent. Chronic ER stress, oxidative stress, or excessive IP_3_R activity remodels MAM architecture and induces ER‐to‐mitochondria Ca^2+^ flux, which results in mitochondrial Ca^2+^ overload, opening of the permeability transition pore (mitochondrial permeability transition pore), mitochondrial depolarization, and apoptotic or necrotic cell death.^55^ Importantly, UPR transducers directly regulate this axis: IRE1α acts as a structural determinant of IP_3_R positioning at the MAMs independently of its transcriptional outputs, thereby tuning basal mitochondrial Ca^2+^ uptake and bioenergetics.^17^

#### 3.1.2 Mitochondrial dynamics axis: ER‐defined fission and stress‐induced fragmentation

ER–mitochondria contact sites act as spatial landmarks for mitochondrial division. High‐resolution, live‐cell imaging revealed that ER tubules wrap around mitochondria and preconstrict them at future fission sites before the recruitment of the dynamin‐related GTPase DRP1.^56^ This process is coordinated, at least in part, by actin polymerization, which is driven by the ER‐localized formin INF2 and myosin II. These proteins mechanically constrict mitochondria and facilitate DRP1 oligomerization on the outer mitochondrial membrane.^57^ In contrast, mitochondrial fusion is regulated by MFN1/2 on the outer membrane and OPA1 on the inner membrane. MFN2 has a dual functional role, acting as a mitochondrial fusion protein and, in specific contexts, as an ER–mitochondria tether. MFN2 disruption alters contact site architecture, Ca^2+^ transfer, and mitochondrial morphology; however, its precise tethering role appears cell‐type and context dependent.^58^, ^59^, ^60^ During chronic metabolic or oxidative stress, pathological activation of DRP1 combined with impaired fusion shifts the balance toward mitochondrial fragmentation, respiratory chain inefficiency, and enhanced sensitivity to apoptotic stimuli. These features are consistently observed in neurodegenerative and ophthalmic disease models.^3^, ^61^, ^62^, ^63^, ^64^

#### 3.1.3 Cell‐death and quality‐control axis: Decision nodes at the ER–mitochondria interface

In addition to metabolic regulation, MAMs act as decision‐making platforms for cell fate by integrating Ca^2+^ signaling, mitochondrial dynamics, and the apoptotic machinery. The BAP31–FIS1 complex at the MAMs coordinates Ca^2+^ release from the ER with mitochondrial fission and caspase‐8 activation, which links ER stress directly to mitochondrial outer membrane permeabilization.^65^ Persistent ER stress recruits pro‐apoptotic BCL‐2 family members (Bcl‐2‐associated X protein/Bcl‐2‐antagonist killer) to MAM‐proximal mitochondria, which amplifies cytochrome c release and apoptotic cascade activation.^66^, ^67^, ^68^

In parallel, intact ER–mitochondria contacts are required for autophagosome formation and mitophagy initiation, with MAMs acting as privileged sites for autophagic membrane nucleation. The disruption of contact integrity impairs mitophagic flux, enabling damaged mitochondria to accumulate and exacerbate oxidative stress.^69^, ^70^ Taken together, dysfunction of this axis converts MAMs from adaptive stress‐buffering hubs into amplifiers of mitochondrial damage and regulates cell death.

### 3.2 Pathological remodeling of MAMs under chronic stress

During sustained metabolic, oxidative, or proteotoxic stress, ER–mitochondria contact sites undergo rapid and compositional remodeling, rather than existing as static interfaces. High‐resolution imaging and single‐molecule tracking revealed that contact sites consist of dynamic subdomains and can expand or reorganize to accommodate changing physiological demands, such as nutrient stress.^71^ Consistent with this plasticity, UPR transducers directly contribute to MAM homeostasis through non‐canonical structural functions: IRE1α acts as a scaffold to control InsP_3_R distribution at the MAMs and tunes mitochondrial Ca^2+^ uptake and bioenergetics,^17^ whereas PERK localizes to contact sites and sustains ER–mitochondria apposition to enhance reactive oxygen species (ROS) propagation during chronic stress.^15^ Simultaneously, stress‐responsive post‐translational regulation of tethering complexes (e.g., VAPB–PTPIP51) reshapes coupling strength and Ca^2+^ transfer, providing a route through which disease‐linked signals, including kinase activation, disrupt ER–mitochondria communication.^72^, ^73^ Oxidative lipid stress can trigger minute‐scale expansion of ER–mitochondria contact interfaces and bias these sites toward pro‐oxidant outcomes, such as mitochondrial ROS amplification and fission, which implicates pathological MERC remodeling as an early and potentially reversible amplifier that lowers the threshold for mitochondrial failure and regulated cell death.^74^, ^75^

## 4 PATHOLOGICAL IMPLICATIONS OF ER–MITOCHONDRIA CROSSTALK IN OPHTHALMIC DISEASES

### 4.1 Cataract: The oxidative stress–ER stress–mitochondrial dysfunction triad

**FIGURE 2:**
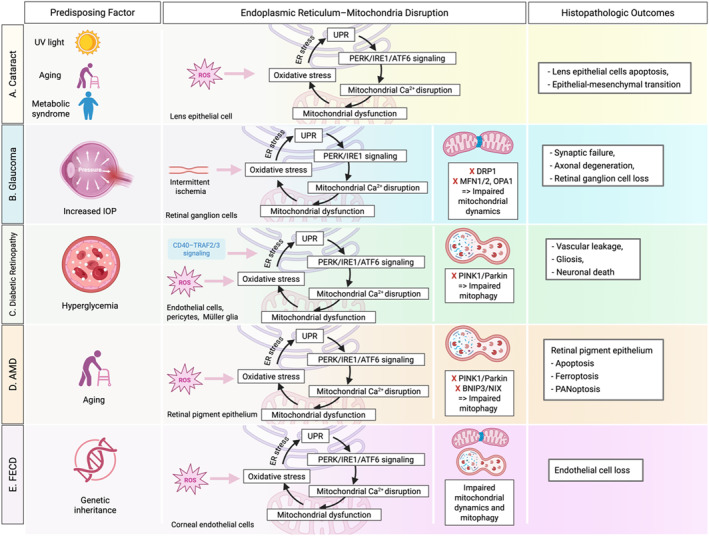
The central role of ER–mitochondria crosstalk in the pathogenesis of ocular conditions: cataract (A), glaucoma (B), diabetic retinopathy (C), age‐related macular degeneration (D), and Fuchs endothelial corneal dystrophy (E). Chronic stressors (e.g., UV light, aging, elevated intraocular pressure, hyperglycemia, and genetic predisposition) induce oxidative stress and ROS accumulation in vulnerable cell types. This triggers ER stress and activation of the unfolded protein response branches (e.g., PERK/eIF2α/ATF4/CHOP, IRE1/XBP1, ATF6), which results in mitochondrial Ca^2+^ overload and dysfunction through dysregulated mitochondria‐associated ER membranes. Impaired mitochondrial dynamics (e.g., excess DRP1‐mediated fission, reduced MFN1/2/OPA1 fusion), defective mitophagy (e.g., PINK1/Parkin or BNIP3/NIX pathways), and bioenergetic collapse amplify ROS and inflammation, thus driving disease‐specific outcomes. ATF4, activating transcription factor 4; ATF6, activating transcription factor 6; CHOP, the C/EBP homologous protein; eIF2α, eukaryotic initiation factor 2 alpha; ER, endoplasmic reticulum; MFN, mitofusin; PERK, protein kinase RNA‐like endoplasmic reticulum kinase; ROS, reactive oxygen species; UV, ultraviolet; XBP1, X‐box‐binding protein‐1.

Cataract is characterized by the loss of proteostasis and redox balance in LECs (Figure 2A). While the general cascades of UPR activation and MAM destabilization are summarized in Table 1, specific evidence in LECs highlights a unique vulnerability to organelle‐interface disruption.^9^, ^10^, ^11^ At the organelle level, stress‐induced MAM destabilization is associated with mitochondrial Ca^2+^ overload and ROS amplification, a paradigm increasingly invoked as a causal mechanism for LEC apoptosis.^10^, ^11^, ^12^

**TABLE 1:** ER–mitochondria crosstalk landscape across major ophthalmic diseases.

Disease	Primary stressor(s)	Key UPR/MAM alterations	Mitochondrial phenotype	Cell fate outcome	Therapeutic strategy
Cataract	UV, aging, metabolic/oxidative stress	PERK–eIF2α–ATF4–CHOP, IRE1/XBP1; MAM Ca^2+^ overload	ΔΨm depolarization, ATP deficit, ROS amplification	LEC apoptosis, EMT, fibrosis	Chemical chaperones (PBA, TUDCA), PERK/ATF6 modulation, MAM stabilization
Glaucoma	Elevated IOP, ischemia/reperfusion	PERK–CHOP activation; MAM Ca^2+^ dysregulation	DRP1‐mediated fission, mitophagy impairment, mtDNA damage	RGC apoptosis, axonal energy failure	DRP1 inhibition, PERK inhibitors, MAM‐targeted Ca^2+^ buffering
Diabetic retinopathy	Hyperglycemia, AGEs, oxidative stress	IRE1α scaffolding at MAMs; PERK activation	MAM proteome remodeling, impaired PINK1/Parkin mitophagy	Endothelial/neuronal death, barrier breakdown	PRDX4 reinforcement, PERK inhibition, mitophagy restoration
AMD	Chronic oxidative stress, lipofuscin (A2E), aging	ER–mitochondria Ca^2+^ flux dysregulation, lipid transfer defects	mtDNA damage, PINK1/Parkin failure, fission/fusion imbalance	RPE atrophy, PANoptosis, senescence	MAM stabilization, mitophagy enhancement, mitochondrial transplantation
FECD	Oxidative/proteotoxic stress	PERK–eIF2α–p38 MAPK–CHOP axis; MAM disruption	ΔΨm loss, increased fission, autolysosomal dysfunction	CEC apoptosis, attrition	p38 MAPK inhibition, MitoQ, mitochondrial supplementation

Abbreviations: ATF4, activating transcription factor 4; ATF6, activating transcription factor 6; ATP, adenosine triphosphate; CHOP, the C/EBP homologous protein; eIF2α, eukaryotic initiation factor 2 alpha; ER, endoplasmic reticulum; IOP, intraocular pressure; IRE1α; inositol‐requiring enzyme 1α; MAM, mitochondria‐associated membrane; MAPK, mitogen‐activated protein kinase; PERK, protein kinase RNA‐like endoplasmic reticulum kinase; ROS, reactive oxygen species; UPR, unfolded protein response; UV, ultraviolet; XBP1, X‐box‐binding protein‐1.

The involvement of ER stress is supported by robust activation of BiP/GRP78 and CHOP in models such as the sodium‐selenite rat.^76^ Beyond apoptosis, ER stress has been implicated in the regulation of the EMT, which underlies anterior subcapsular cataract and posterior capsular opacification.^77^ Genetic models provide the most direct evidence of organelle crosstalk; for instance, αA‐crystallin mutations (R49C and Y118D) engage the ER–mitochondria axis, contributing to nuclear cataract, and suggesting that single‐gene misfolding may facilitate systemic organelle failure.^78^, ^79^ Human data mirror these findings, with aged donor lenses and aqueous humor proteomics showing enriched ER‐stress and metabolic dysfunction pathways.^80^, ^81^

Recent translational efforts focus on disrupting the ER‐stress–fibrosis axis. Chemical chaperones, including 4‐phenylbutyrate (PBA) and tauroursodeoxycholic acid (TUDCA), have been shown to attenuate high‐glucose–induced EMT in human LECs.^22^ A key mechanistic highlight is the ATF6–SNAI1 self‐amplifying loop; its inhibition via AAV‐shATF6 or melatonin attenuates fibrotic changes, positioning ATF6 as a specific druggable node at the ER–mitochondria interface.^24^ Additional pharmacological leads, such as SESN2 up‐regulation and mitochondria‐targeted peptides (e.g., elamipretide), offer a roadmap for non‐surgical therapy by safeguarding ATP sufficiency and avoiding CHOP‐dominated apoptosis.^25^, ^82^, ^83^, ^84^, ^85^ Ultimately, cataract represents a prototypical proteostasis‐driven disorder where maladaptive UPR and MAM remodeling are closely linked to terminal fibrotic remodeling.^9^, ^12^ Taken together, cataract represents a prototypical proteostasis‐driven ER–mitochondria disorder, in which maladaptive UPR activation in LECs precedes mitochondrial dysfunction and promotes cell death and fibrotic remodeling.

### 4.2 Glaucoma: ER–mitochondria dysregulation in retinal ganglion cell degeneration

Glaucoma is a progressive optic neuropathy marked by the selective vulnerability of RGCs, whose long axons and high metabolic demand render them exquisitely dependent on efficient mitochondrial ATP production and QC^18^, ^19^ (Figure 2B). Beyond the general UPR signaling summarized in Table 1, the glaucomatous retina undergoes a critical shift where sustained stress converts adaptive responses into pro‐apoptotic programs, resulting in synaptic failure and axonal degeneration.^9^, ^20^ At the ER–mitochondria interface, MAMs coordinate Ca^2+^ transfer and lipid exchange. Destabilization of the MAMs during glaucomatous stress results in mitochondrial Ca^2+^ overload, ROS amplification, and the permeability transition, which reduces the threshold for RGC apoptosis.^21^ A second, tightly linked axis is mitochondrial dynamics, in which pathologic activation of DRP1‐mediated fission with impaired MFN1/2‐ and OPA1‐dependent fusion fragments the mitochondrial network. This reduces respiratory capacity and sensitizes RGCs to death.^18^, ^26^, ^27^

Evidence from rodent models suggests that increasing IOP induces mitochondrial fission and ultrastructural disruption in RGC axons prior to cell loss, positioning organelle reprogramming as an early contributing event.^28^ Actions such as the genetic or pharmacologic inhibition of DRP1 have been shown to preserve mitochondrial integrity and rescue RGC function, supporting the hypothesis that excessive fission is a targetable bottleneck in disease progression.^27^ Recent single‐cell analyses further implicate a broader failure of QC, including mtDNA damage and defective mitophagy.^19^ Importantly, the ER–mitochondria unit acts as an “integrated operative locus” rather than two independent organelles, where ER stress and mitochondrial failure maybe mutually reinforced via MAMs.^21^ For example, TrkB pathway augmentation reveals that preserving axonal transport is closely linked to the maintenance of mitochondrial output, further linking organelle crosstalk to functional survival.^29^

Human postmortem and surgical data are consistent with these preclinical patterns, showing widespread mitochondrial compromise and increased ER‐stress markers in the optic nerve head and inner retina.^19^, ^30^ Two complementary therapeutic strategies are emerging. First, tuning ER stress: small‐molecule PERK inhibitors (e.g., LDN‐0060609) suppress maladaptive UPR outputs in human trabecular meshwork and astroglial models associated with increased pressure and neuroinflammation. This indicates a translatable entry point upstream of CHOP‐driven death programs.^31^, ^32^ Second, stabilizing mitochondrial morphology through DRP1 inhibitors or enhancing mitophagy is associated with vision protection in preclinical systems.^27^, ^86^ Finally, the MAMs perspective suggests an integrated screen for candidates that normalize ER–mitochondria calcium homeostasis and lipid transfer. By preserving the fidelity of this physical interface, such agents could reduce ER stress, improve ATP supply, and increase the injury threshold of RGCs, even when IOP is medically controlled but progression continues.^21^ In summary, the disruption of ER–mitochondria signaling in RGCs represents a primary mechanism associated with axonal energy failure and progressive neurodegeneration.

### 4.3 DR: Metabolic stress linking ER stress and mitochondrial pathways

Chronic hyperglycemia in the diabetic retina initiates a self‐reinforcing loop of oxidative stress, UPR activation, and MAM‐mediated mitochondrial collapse (Figure 2C). MAMs serve as a site for Ca^2+^ and lipid exchange. Under metabolic stress, altered MAM composition is associated with changes in ER‐to‐mitochondria Ca^2+^ transfer, potentially contributing to the permeability transition and bioenergetic collapse. This mechanism is supported by proteomics in diabetic retinas and mechanistic studies showing IRE1α′s scaffolding role at the MAMs.^17^, ^87^ Furthermore, hyperglycemia disrupts the PINK1/Parkin‐mediated mitophagy pathway, compounding ROS and inflammasome signaling across vascular, glial, and neuronal compartments.^88^, ^89^, ^90^, ^91^

Illustrative mechanistic studies highlight specific molecular nodes that govern this organelle crosstalk. Retinal MAM proteomics reveal extensive interface remodeling involving over 180 differentially regulated proteins.^87^ In Müller cells, the ER‐resident antioxidant PRDX4 represents a key node. PRDX4 deficiency is linked to accelerated STZ‐induced retinal pathology, whereas PRDX4 overexpression is associated with reduced reactive gliosis, apoptosis, ER stress, oxidative stress, and mitochondrial dysfunction. These findings suggest that strengthening ER redox control may support mitochondrial stability in the diabetic retina.^33^ Beyond traditional UPR signaling, immune co‐stimulation via the CD40–TRAF2/3 axis intersects with ER stress to drive vascular leakage, linking inflammatory tone to organelle‐mediated barrier failure.^92^ Additionally, targeting the PERK branch with inhibitors like GSK2606414 or modulating NR2F2 has been shown to improve retinal structure and function in preclinical models of ischemic and microvascular injury.^34^, ^93^

Human biomarker studies increasingly mirror the experimental results. Quantitative proteomics of aqueous humor across DR stages (non‐proliferative diabetic retinopathy → proliferative diabetic retinopathy) has revealed stage‐dependent shifts in inflammation, complement, oxidative stress, and metabolic networks. This indicates that redox/ER‐stress pathways are engaged in vivo and are scalable as fluid biomarkers.^94^, ^95^ Clinical observations of increased GRP78 and mitochondrial functional markers in patient fluids correlate with VEGF levels and macular edema, supporting the involvement of these organelle axes in human disease.^17^, ^33^, ^88^, ^96^, ^97^ Integrating these mechanism‐based approaches with standard anti‐VEGF therapy may facilitate earlier detection and more effective intervention for DR as a metabolically driven ER–mitochondria stress syndrome.^94^ Thus, DR reflects a metabolically driven ER–mitochondria stress syndrome, in which chronic hyperglycemia induces sustained MAM remodeling and impaired mitophagy, amplifying oxidative stress, inflammation, and vascular–neural dysfunction.

### 4.4 Age‐related macular degeneration: Organelle crosstalk driving RPE senescence and atrophy

In AMD, the RPE endures chronic oxidative and proteotoxic stress, which activates the UPR and is associated with the progressive coupling of ER dysfunction to mitochondrial failure^9^ (Figure 2D). While the basic UPR‐MAM signaling framework is summarized in Table 1, the RPE exhibits unique pathomechanisms such as A2E‐induced phototoxicity and ZBP1‐mediated PANoptosis, an integrated inflammatory cell death pathway that coordinates key features of apoptosis, pyroptosis, and necroptosis.^12^, ^98^, ^99^ Concurrently, a decline in PINK1/Parkin and BNIP3/NIX‐dependent mitophagy is associated with impaired the removal of damaged mitochondria, potentially contributing to a feed‐forward loop of ROS and inflammatory signaling.^100^, ^101^

Regarding multi‐omic and model systems of the ER–mitochondria axis, iPSC‐derived RPE from AMD donors (and those harboring risk variants) show impaired oxidative phosphorylation and an increased susceptibility to mitochondrial toxins. This provides a platform in which mitochondria‐targeted drugs can restore bioenergetics, which functionally validates mitochondria as a target in AMD.^102^ Mechanistic studies using RGR‐deletion or PGC‐1‐related mouse models associate autolysosomal defects and mitophagy failure with RPE degeneration in vivo.^103^, ^104^ A complementary data map revealed how aging and altered redox status reshape mitochondrial dynamics (DRP1/MFN/OPA1), crosstalk with complement, and regulate cell death programs during geographic atrophy. This situates organelle stress early in the AMD cascade.^105^, ^106^

Human donor studies consistently document an organelle‐linked bioenergetic crisis in AMD. Mitochondrial DNA damage is significantly accentuated in RPE from carriers of the complement factor H Y402H risk variant, aligning genetic susceptibility with bioenergetic failure.^107^, ^108^ Beyond cell‐intrinsic defects, mitochondria‐associated autoantibodies are enriched in AMD and correlate with disease mechanisms, suggesting an immune amplification layer that intersects with organelle stress.^109^ Treatment strategies involve: (i) UPR tuning to avoid CHOP‐dominated maladaptation, while sustaining proteostasis,^9^ (ii) MAM stabilization/Ca^2+^ control to preserve ATP supply and limit ROS‐inflammation coupling,^12^ and (iii) mitochondrial quality‐control restoration (enhancing PINK1/Parkin or BNIP3/NIX mitophagy) to eliminate dysfunctional organelles.^100^ Translational momentum includes mitochondria‐targeted drug screening in iPSC‐RPE and early‐stage concepts, such as mitochondrial transplantation for retinal degenerations, which supports the feasibility of reinforcing the ER–mitochondria unit pharmacologically or biologically in human tissue models.^102^, ^110^ Integrating these organelle‐targeted strategies with high‐resolution imaging and fluid biomarkers will enable the earlier detection of RPE failure and guide the next generation of AMD combination therapies.^105^

### 4.5 Fuchs endothelial corneal dystrophy: ER–mitochondria imbalance in corneal endothelial cell loss

FECD is hallmarked by the progressive attrition of mitochondria‐rich, minimally proliferative CECs^111^ (Figure 2E). Chronic oxidative and proteotoxic stress in these cells triggers a transition from adaptive proteostasis to metabolic rewiring and apoptosis.^111^, ^112^ While the canonical UPR branches and general MAM‐mediated Ca^2+^ flux dysregulation are summarized in Table 1, the FECD phenotype is uniquely defined by a rapid loss of mitochondrial membrane potential (ΔΨm) and ATP sufficiency.^112^, ^113^

A hierarchical signaling study indicated that p38 mitogen‐activated protein kinase (MAPK) is downstream of PERK–eIF2α, yet upstream of ATF4–CHOP. Inhibiting p38 preserves ΔΨm and suppresses apoptosis in multiple ER‐stress scenarios (TGF‐β–conditioned FECD cells, thapsigargin, MG‐132). This suggests a potential signaling switch involved in the transition of adaptive UPR into death signaling in the corneal endothelium.^114^ Besides signaling, mitochondrial QC is a central failure point. Reviews and experimental studies have documented mitophagy/autophagy dysregulation in FECD, suggesting that the impaired clearance of dysfunctional mitochondria compounds ROS and ER stress.^115^ In Slc4a11−/− models, mitochondrial ROS has been shown to lie upstream of ER stress, reinforcing the concept that mitochondrial stabilization is a primary requirement for maintaining the corneal endothelial pump.^116^

Translational strategies are coalescing around restoring organelle fidelity. Tuning the UPR through p38 MAPK effectors or PERK‐proximal outputs may help rebalance ER signaling away from CHOP‐dominated death.^114^ On the mitochondrial front, the antioxidant MitoQ and NAD^+^‐precursor support have demonstrated efficacy in protecting CECs from oxidative and ultraviolet B‐induced injury.^117^, ^118^ Most notably, the exogenous supplementation of mitochondria (mitochondrial transplantation) into FECD patient explants has been shown to reduce oxidative stress and restore ΔΨm, providing a proof‐of‐concept for organelle‐based biological rescue.^119^, ^120^ Targeting the ER–mitochondria interface to normalize MAM‐mediated flux remains a promising, mechanism‐based strategy to preserve the minimally proliferative CEC population.^119^, ^121^

## 5 TARGETING ER–MITOCHONDRIA CROSSTALK IN OPHTHALMIC DISEASES

**FIGURE 3:**
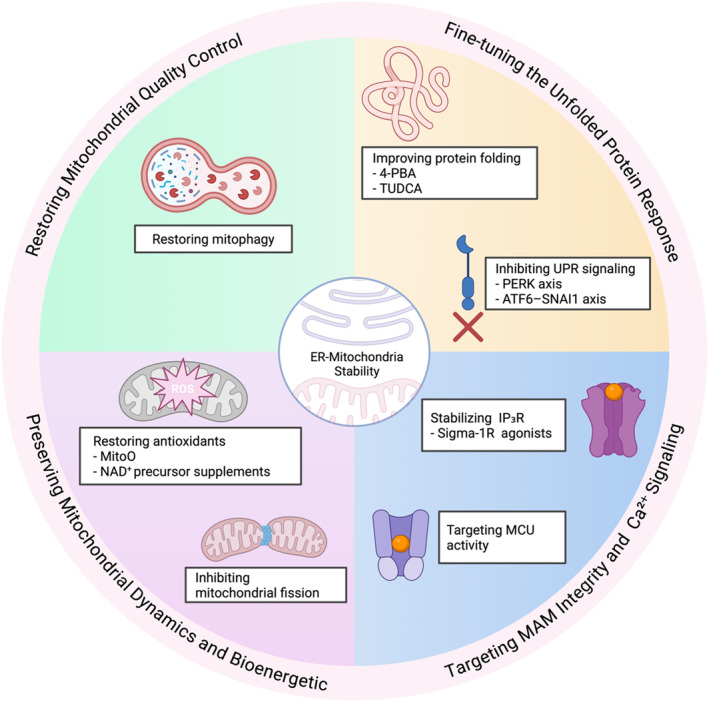
Therapeutic approaches for targeting ER–mitochondria interact to restore mitochondrial quality control, dynamics, bioenergetics, and Ca^2+^ signaling in retinal and corneal disorders. Such interventions include fine‐tuning the UPR, stabilizing MAMs, modulating MCU activity, preserving mitochondrial dynamics, and inducing mitophagy to disrupt stress cycles and protect ocular cells. AMD, age‐related macular degeneration; ER, endoplasmic reticulum; MAM, mitochondria‐associated membrane; MCU, mitochondrial calcium uniporter; UPR, unfolded protein response.

Building on the mechanistic evidence summarized in Sections 2, 3, 4, it is clear that ER stress, MAM remodeling, Ca^2+^ dysregulation, and mitochondrial dysfunction are not isolated pathological events in ophthalmic diseases. Instead, they form a tightly coupled stress network that amplifies cellular vulnerability in ocular tissues. This convergence provides a rationale for therapeutic strategies that target the ER–mitochondria axis itself, rather than focusing exclusively on downstream manifestations, such as lens opacity, increased IOP, neovascularization, or endothelial cell loss. Emerging interventions may be broadly categorized into four mechanistically aligned groups: (i) modulation of maladaptive UPR signaling, (ii) stabilization of MAM architecture and Ca^2+^ flux, (iii) preservation of mitochondrial dynamics and bioenergetics, and (iv) restoration of mitochondrial QC through mitophagy (Figure 3).

### 5.1 Fine‐tuning the UPR to avoid maladaptive ER stress

As discussed in Sections 2 and 4, sustained activation of the PERK–eIF2α–ATF4–CHOP axis represents a pro‐apoptotic switch in LECs, RGCs, retinal Müller cells, RPE, and CECs during conditions of chronic stress.^9^, ^112^ Therefore, therapeutic strategies aimed at attenuating maladaptive UPR outputs, while preserving adaptive proteostasis, have gained interest. Chemical chaperones, such as 4‐phenylbutyrate (4‐PBA) and TUDCA, reduce ER stress by enhancing protein folding capacity, and they exert protective effects in models of cataract‐related EMT, corneal endothelial degeneration, and metabolic retinal stress.^22^, ^112^

Beyond global chaperoning, pathway‐selective modulation of UPR sensors offers greater precision. The inhibition of PERK signaling ameliorates RGC death in glaucomatous models^31^, ^32^ and reduces pathological angiogenesis and neuronal loss in ischemic and DR paradigms.^93^ Similarly, targeting ATF6‐dependent transcriptional loops, such as the ATF6–SNAI1 axis implicated in lens fibrosis, attenuates EMT and capsular opacification, which highlights the disease‐specific utility of branch‐selective UPR intervention.^24^ These results support a therapeutic paradigm in which UPR signaling is not globally suppressed, but dynamically rebalanced to prevent ER stress from causing mitochondrial failure.

### 5.2 Targeting MAM integrity and ER–mitochondria Ca^2+^ signaling

MAMs constitute the physical and functional hub of ER–mitochondria crosstalk, as discussed in Section 3. They regulate Ca^2+^ transfer, lipid metabolism, and apoptotic signaling.^3^, ^12^ Disruption of MAM architecture through altered MFN2, PACS2, VAPB–PTPIP51, or InsP_3_R–GRP75–VDAC1 complexes has been implicated in glaucoma,^21^ DR,^87^ AMD,^99^ and FECD.^113^ Restoring balanced ER‐to‐mitochondria Ca^2+^ flux represents an effective strategy to prevent mitochondrial Ca^2+^ overload, permeability transition pore opening, and ROS amplification. Although pharmacological stabilizers of MAMs are still in early development, indirect modulation of Ca^2+^ handling has shown promise. Sigma‐1 receptor agonists, which stabilize InsP_3_R function at the MAMs and prolong adaptive mitochondrial Ca^2+^ uptake, exert neuroprotective effects in retinal neurons and are mechanistically consistent with preserving ER–mitochondria coupling.^122^ Similarly, targeting excessive mitochondrial Ca^2+^ influx by modulating mitochondrial calcium uniporter (MCU) activity or upstream ER Ca^2+^ release may prevent stress‐induced bioenergetic collapse in retinal and corneal cells.^17^, ^123^ These approaches are consistent with the evidence that MAM dysfunction represents an early and reversible event in ocular disease progression.

### 5.3 Preserving mitochondrial dynamics and bioenergetic homeostasis

As discussed in Sections 3.1 and 4, pathological remodeling of mitochondrial dynamics, which is characterized by excessive DRP1‐mediated fission and impaired MFN/OPA1‐dependent fusion, contributes directly to neuronal and epithelial vulnerability in glaucoma,^26^, ^27^, ^28^ AMD,^105^, ^106^ and FECD.^112^ Pharmacological or genetic inhibition of DRP1 preserves mitochondrial network integrity, maintains ATP production, and promotes RGC survival and axonal function in experimental glaucoma.^27^ These results support mitochondrial dynamics as a therapeutic target downstream of ER–mitochondria stress signaling.

Complementary strategies focus on reinforcing mitochondrial bioenergetics and redox capacity. Mitochondria‐targeted antioxidants, such as MitoQ, can restore mitochondrial membrane potential, reduce cytochrome c release, and enhance cell survival in corneal endothelial and retinal stress models.^116^, ^117^ Similarly, NAD^+^ precursor supplementation promotes mitochondrial resilience against ultraviolet‐ and oxidative‐stress‐induced damage in corneal and retinal tissues, thus dampening ER stress propagation.^118^ Taken together, these strategies indicate that preserving mitochondrial function can interrupt the vicious cycle linking ER stress to cell degeneration.

### 5.4 Restoring mitochondrial quality control via mitophagy

Impaired mitochondrial QC is a recurring theme among ophthalmic diseases, particularly in DR,^90^, ^91^ AMD,^103^, ^104^ glaucoma,^86^ and FECD,^119^ in which defective PINK1/Parkin‐ or BNIP3/NIX‐mediated mitophagy results in the accumulation of dysfunctional mitochondria. Because of the intimate coupling between mitophagy, ER stress resolution, and MAM signaling, therapeutics aimed at restoring mitophagic flux have emerged as a key downstream component of ER–mitochondria–targeted intervention.

In metabolic and degenerative retinal diseases, such as DR and AMD, evidence indicates that impaired mitophagy amplifies mitochondrial ROS production, inflammasome activation, and inflammatory cell death, thereby reinforcing ER stress and MAM dysfunction. Pharmacological or genetic enhancement of mitophagy can reduce oxidative injury, suppress inflammatory signaling, and stabilize mitochondrial bioenergetics in retinal endothelial cells, Müller glia, and RPE, which support mitophagy as a disease‐modifying target in preclinical models.^90^, ^91^, ^103^, ^104^ However, translation to these diseases is constrained by challenges in cell‐type‐specific delivery and the need to preserve mitochondrial mass in tissues with high energetic demand. This highlights the importance of stage‐ and context‐dependent modulation, rather than indiscriminate activation of mitochondrial clearance.

In glaucoma, mitophagy dysfunction has been implicated in RGC vulnerability, in which impaired clearance of damaged mitochondria exacerbates bioenergetic failure and axonal degeneration.^18^, ^19^ Although direct therapeutic manipulation of mitophagy in glaucoma is at an early experimental stage, studies suggest that restoring mitochondrial QC may synergize with interventions targeting mitochondrial dynamics and ER stress to preserve RGC survival during the early stages of disease.^86^

Notably, the strongest translational evidence for targeting mitochondrial QC is in FECD. Studies using exogenous mitochondrial supplementation in patient‐derived corneal endothelium revealed that direct restoration of mitochondrial capacity reduces oxidative stress, normalizes mitochondrial membrane potential, and rebalances excessive mitophagy in human tissues ex vivo.^117^, ^119^ These results establish organelle‐level mitochondrial intervention as a feasible treatment strategy and position FECD as a model disease for translating ER–mitochondria–centered therapies toward clinical application.

Taken together, these observations indicate that while mitophagy restoration represents a shared therapeutic axis across multiple ophthalmic diseases, optimal intervention differs by tissue context and disease stage. FECD provides a near‐clinical paradigm for direct mitochondrial rescue, whereas DR, AMD, and glaucoma may benefit from more nuanced approaches that integrate mitophagy modulation with upstream ER stress control and mitochondrial dynamics stabilization.

## 6 TOWARD INTEGRATED, MECHANISM‐BASED AND CLINICALLY PRIORITIZED THERAPIES IN OPHTHALMOLOGY: FROM DESCRIPTIVE ORGANELLE STRESS TO STAGE‐SPECIFIC THERAPEUTICS

**FIGURE 4:**
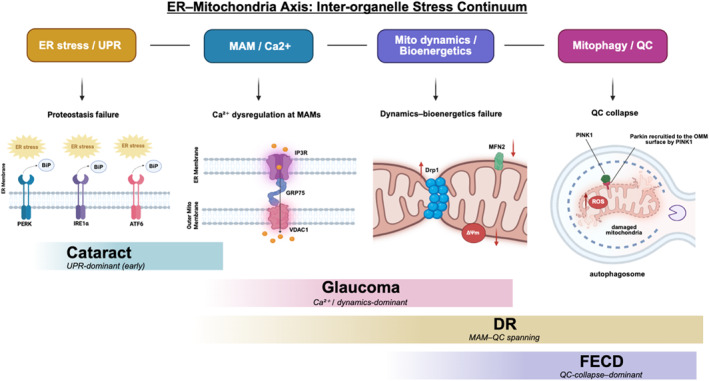
Node‐dominant, stage‐dependent framework of ER–mitochondria crosstalk in ophthalmic diseases. The ER–mitochondria axis is displayed as a continuum of inter‐organelle stress states, which include UPR‐mediated proteostasis failure, dysregulated Ca^2+^ signaling at mitochondria‐associated membranes, mitochondrial dynamics and bioenergetic failure, and the collapse of mitochondrial quality control. Ophthalmic diseases are characterized by the dominance of distinct pathological nodes along this axis, which define a spectrum rather than discrete disease entities. This framework provides a basis for designing stage‐specific and mechanism‐oriented therapeutics [Created in BioRender. Shawn, M. (2026), https://BioRender.com/hutvbmv.]. ER, endoplasmic reticulum; UPR, unfolded protein response.

In this review, the evidence described reframes of ophthalmic diseases as disorders of inter‐organelle communication, rather than isolated failures of single cellular compartments. ER stress, MAM remodeling, Ca^2+^ dysregulation, and mitochondrial dysfunction form a hierarchically organized stress continuum that regulates cell fate across diverse ocular tissues. As a result, we propose a node‐dominant, stage‐dependent framework of ER–mitochondria crosstalk, in which distinct pathological nodes are predominant at different disease stages and across diverse ophthalmic tissues. This node‐dominant, stage‐dependent framework is summarized in Figure 4. ER–mitochondria crosstalk emerges not merely as a convergent downstream consequence of disease, but as an upstream, modifiable axis that integrates proteostasis, bioenergetics, redox signaling, and cell death decisions.^9^, ^12^ Thus, we propose that ophthalmic diseases can be mechanistically stratified by their dominant failure points along the ER–mitochondria axis, which provides a rationale for disease‐ and stage‐specific therapeutic prioritization.

Distinct ophthalmic diseases are characterized by different dominant nodes along the ER–mitochondria axis, rather than a uniform pathogenic mechanism. In cataract, maladaptive UPR signaling and proteostasis are disrupted in LECs, which precede mitochondrial failure and the EMT. This positions UPR modulation as an early disease‐modifying strategy.^76^, ^78^, ^124^ In glaucoma, biomechanical and ischemic stress preferentially disrupts ER–mitochondria Ca^2+^ transfer and mitochondrial dynamics in RGCs, which results in axonal energy failure and neurodegeneration.^18^, ^21^, ^26^, ^27^ In DR, chronic metabolic stress drives sustained MAM remodeling and mitophagy impairment across vascular, glial, and neuronal compartments, which creates a feed‐forward loop between ER stress, mitochondrial dysfunction, and inflammation.^87^, ^88^, ^89^, ^90^ In contrast, FECD represents a paradigm of ER‐stress, which is driven by mitochondrial collapse in a minimally regenerative tissue, and PERK–p38–CHOP signaling and defective mitochondrial QC directly affect cell survival.^112^, ^114^, ^115^, ^119^

Disease‐node stratification has direct translational implications. It argues against indiscriminate suppression of ER stress or global enhancement of mitochondrial turnover, and instead, supports stage‐ and tissue‐specific prioritization of therapeutic targets. During early and intermediate disease stages, when ER stress activation and MAM remodeling are partially reversible, selective tuning of maladaptive UPR outputs, particularly the PERK–eIF2α–ATF4–CHOP axis, can prevent mitochondrial injury. At later stages, when mitochondrial fragmentation, bioenergetic failure, and defective mitophagy dominate the pathology, interventions aimed at stabilizing mitochondrial dynamics, reinforcing redox capacity, and restoring mitochondrial QC are more likely to be effective. Importantly, excessive or prolonged suppression of stress responses may be deleterious, which emphasizes the need for tunable, rather than binary modulation of ER–mitochondria signaling.^14^, ^125^

From a clinical perspective, ophthalmology offers unique advantages for translating ER–mitochondria‐targeted therapies. Local drug delivery minimizes systemic exposure, whereas the aqueous and vitreous humor provide accessible biofluids for biomarker‐guided patient stratification. Molecular indicators of ER stress (e.g., GRP78, XBP1s, ATF4, and CHOP) and mitochondrial dysfunction (e.g., mtDNA damage, mitophagy markers) may facilitate the identification of therapeutic windows, when intervention at the ER–mitochondria interface is most effective.^9^, ^21^, ^41^

Ophthalmic diseases can be broadly categorized based on their dominant pathological nodes along the ER–mitochondria axis. These include: (i) UPR‐dominant proteostasis failure; (ii) MAM–Ca^2+^ dysregulation–driven signaling imbalance; (iii) mitochondrial dynamics and bioenergetics failure; and (iv) mitochondrial QC collapse–driven degeneration. Together, they define a spectrum of inter‐organelle stress states, rather than discrete disease entities. Therefore, the ER–mitochondria axis not only provides a unifying mechanistic framework for diverse ocular diseases, but also a foundation for designing stage‐specific, combination therapies aimed at preserving cellular homeostasis and long‐term visual function.

## 7 LIMITATIONS, CONTROVERSIES, AND INTEGRATED FUTURE PERSPECTIVES

Mounting evidence positions ER–mitochondria crosstalk as a central regulator of ocular cell fate; however, several limitations constrain interpretation and translation. First, ER–mitochondria signaling is highly cell‐type‐ and context‐dependent among the ocular tissues, and the results derived from isolated models may not be directly generalizable to the heterogeneous and slowly evolving human disease milieu.^126^ Second, most experimental systems provide static or acute snapshots of ER stress, MAM remodeling, Ca^2+^ dysregulation, and mitochondrial dysfunction. Thus, the temporal hierarchy and causal relationships among these events remain incompletely resolved.^127^, ^128^

A major controversy in the field concerns the molecular structure of the interface itself, in particular the role of MFN2. Although many studies have classified MFN2 as the main ER–mitochondria tether,^58^ others suggest it may act as a spacer to prevent excessive proximity.^59^, ^60^ In the context of eye diseases, where precise intercellular Ca^2+^ flux is essential for the survival of RPE or RGC, this debate underscores the fact that changes in MAM integrity are often inferred, rather than selectively manipulated. The technical limitations of traditional electron microscopy further hinder this questioning. Although it provides high resolution, its inherent low throughput and susceptibility to fixation‐induced artifacts may distort the delicate membrane distance. In addition, in human‐derived eye tissues, there is a lack of dynamic live cell imaging data, so the real‐time flux of intercellular communication during chronic degeneration is largely unknown. This highlights the need for spatiotemporally controlled interrogation of ER and mitochondria contact sites in vivo. Technical challenges, including low‐throughput ultrastructural analyses, variability of Ca^2+^ reporters, and contamination‐prone MAM proteomics, further complicate the quantitative assessment of pathological versus adaptive coupling.^127^, ^128^

To overcome these barriers, the field is beginning to adopt emerging toolkits such as proximity labeling (e.g., APEX2, BioID) and advanced super‐resolution microscopy (e.g., stimulated emission depletion microscopy, stochastic optical reconstruction microscopy), which offer high‐fidelity proteomic and structural maps of the interface in its native cellular environment.^129^ The intrinsic duality of UPR and mitochondrial stress responses poses a major treatment challenge. A notable example is the modulation of the PERK branch; while PERK inhibition preserves neurons in some ischemic and pressure‐induced models by preventing CHOP‐driven apoptosis, it may be detrimental in others where basal PERK signaling is required for homeostatic proteostasis.^93^ To navigate this, the field must operationalize the distinction between adaptive and maladaptive UPR with specific molecular criteria. We propose that adaptive coupling is characterized by transient eIF2α phosphorylation and robust XBP1 splicing that restores folding capacity,^128^, ^129^ whereas the maladaptive transition is marked by persistent CHOP induction, TXNIP‐mediated inflammasome activation, and sustained mitochondrial Ca^2+^ overload via VDAC1‐IP3R1 complexes. These constraints reinforce the need for stage‐specific prioritization rather than indiscriminate targeting.^39^, ^125^, ^130^

Building on these limitations and the framework proposed in Section 6, we outline four priority directions for advancing the field from descriptive associations toward mechanism‐based, disease‐stage–anchored targeting of the ER–mitochondria axis.

First, defining disease‐specific MAM signatures will be essential. Systematic comparative profiling of MAM‐resident proteomes, MAM‐localized lipid species, and Ca^2+^‐handling parameters across cataract, glaucoma, DR, AMD, and FECD is needed to distinguish shared from disease‐restricted vulnerabilities, to nominate candidate biomarkers, and to validate the dominant pathological nodes proposed in our framework.

Second, developing in vivo tools to quantify ER–mitochondria coupling dynamics is a critical technical priority. Beyond the proximity‐labeling and super‐resolution approaches discussed above, genetically encoded contact‐site reporters (e.g., split‐GFP‐based contact‐site sensors^131^ and dimerization‐dependent fluorescent sensors) and volumetric or cryo‐electron tomography‐based approaches^132^ will be needed to capture interface dynamics in living ocular tissues, where current evidence remains largely static.

Third, clarifying the temporal evolution of pathological nodes along the ER–mitochondria axis is required to test the central hypothesis of stage‐dependent dominance. Cross‐sectional snapshots should be complemented by longitudinal animal models, stage‐stratified human specimens, and, where feasible, in vivo imaging, integrated with single‐cell and spatial multi‐omics^129^ to map how the dominant failure node shifts across early, intermediate, and advanced disease.

Fourth, exploring stage‐specific and combinatorial therapeutic strategies will translate this framework into the clinic. Interventions—including chemical chaperones, IP3R or MCU modulators, MFN2/PACS2 stabilizers, mitophagy enhancers, and integrated stress response modulators—should be matched to both the dominant pathological node and the disease stage, rather than applied indiscriminately. Rationally designed combinations targeting sequential nodes (e.g., early UPR rebalancing followed by later mitophagy reinforcement) deserve dedicated preclinical evaluation in human‐relevant ocular models.^102^

Pursuing these four directions in parallel will be essential to translate ER–mitochondria‐centered strategies into clinically effective interventions that preserve ocular tissue resilience and visual function. Viewing ophthalmic diseases through the lens of ER–mitochondria communication reframes them from organ‐specific pathologies to disorders of inter‐organelle stress integration, opening new avenues for stage‐specific and mechanism‐based intervention.

## AUTHOR CONTRIBUTIONS

**Luyang Jiang and Gongsang Cuomu**: Investigation; data curation; visualization; writing—original draft. **Sang Jijia and Yumei Yang**: Project administration; methodology; writing—review and editing. **Jufen Liu and Yumin Tao**: Conceptualization; supervision; writing—review and editing. All authors have read and agreed to the published version of the manuscript.

## CONFLICT OF INTEREST STATEMENT

The authors declare no conflicts of interest.

## ETHICS STATEMENT

The authors have nothing to report.

## CONSENT TO PARTICIPATE

The authors have nothing to report.

## CONSENT TO PUBLISH

The authors have nothing to report.

## Data Availability

Data sharing not applicable to this article as no datasets were generated or analyzed during the current study.
